# Internal and External Validation of Machine Learning Models for Predicting Acute Kidney Injury Following Non-Cardiac Surgery Using Open Datasets

**DOI:** 10.3390/jpm14060587

**Published:** 2024-05-30

**Authors:** Sang-Wook Lee, Jaewon Jang, Woo-Young Seo, Donghee Lee, Sung-Hoon Kim

**Affiliations:** 1 Department of Anesthesiology and Pain Medicine, Asan Medical Center, University of Ulsan College of Medicine, Seoul 05505, Republic of Korea; sangwooklee@amc.seoul.kr (S.-W.L.); d220404@amc.seoul.kr (D.L.); 2 Biomedical Engineering Research Center, Biosignal Analysis & Perioperative Outcome Research (BAPOR) Laboratory, Asan Institute for Lifesciences, Seoul 05505, Republic of Korea; jjw2582@aitrics.com (J.J.); seowy@amc.seoul.kr (W.-Y.S.); 3 Department of Anesthesiology and Pain Medicine, Brain Korea 21 Project, University of Ulsan College of Medicine, Seoul 05505, Republic of Korea

**Keywords:** acute kidney injury, machine learning, external validation, non-cardiac surgery, postoperative complications

## Abstract

This study developed and validated a machine learning model to accurately predict acute kidney injury (AKI) after non-cardiac surgery, aiming to improve patient outcomes by assessing its clinical feasibility and generalizability. We conducted a retrospective cohort study using data from 76,032 adults who underwent non-cardiac surgery at a single tertiary medical center between March 2019 and February 2021, and used data from 5512 patients from the VitalDB open dataset for external model validation. The predictive variables for model training consisted of demographic, preoperative laboratory, and intraoperative data, including calculated statistical values such as the minimum, maximum, and mean intraoperative blood pressure. When predicting postoperative AKI, our gradient boosting machine model incorporating all the variables achieved the best results, with AUROC values of 0.868 and 0.757 for the internal and external validations using the VitalDB dataset, respectively. The model using intraoperative data performed best in internal validation, while the model with preoperative data excelled in external validation. In this study, we developed a predictive model for postoperative AKI in adult patients undergoing non-cardiac surgery using preoperative and intraoperative data, and external validation demonstrated the efficacy of open datasets for generalization in medical artificial modeling research.

## 1. Introduction

Acute kidney injury (AKI) is a condition characterized by a rapid decline in kidney function and can be caused by a variety of factors [1]. In particular, postoperative patients are known to be at a higher risk of developing AKI than the average non-surgical hospitalized patient [2]. The occurrence of postoperative AKI not only prolongs the patient’s hospitalization duration but also leads to escalated healthcare expenses [3,4]. Furthermore, postoperative AKI can impact the long-term outcome for surgical patients by elevating postoperative morbidity and mortality rates, potentially leading to the development of chronic kidney disease in the future [5,6,7]. Predicting the occurrence of postoperative AKI before surgery enables the allocation of adequate postoperative medical resources to surgical patients, ultimately enhancing patient outcomes and mitigating healthcare costs [8,9]. However, to date, a systematic approach for predicting postoperative AKI in patients undergoing non-cardiac surgery has not been well established. We intend to achieve this by developing a predictive model using machine learning (ML) techniques, which have recently been actively applied in the medical field. Whereas previous artificial intelligence (AI) studies have focused on predicting post-surgery AKI, most relied on data from single institutions, which limited their generalizability [9,10,11,12]. To overcome this limitation, our study aimed to explore the model’s feasibility and utility across different institutions by externally validating it using an openly accessible dataset. In medical AI research, the utilization of open datasets for external validation is a crucial aspect of model development, offering a highly practical and viable alternative. However, because of the challenges associated with sharing and accessing medical data over different time periods, obtaining external datasets for validation remains a formidable task. Therefore, we anticipate that research focused on leveraging open datasets for external model validation will continue to thrive in the future.

## 2. Materials and Methods

### 2.1. Ethical Statement and Study Data

**Figure 1:**
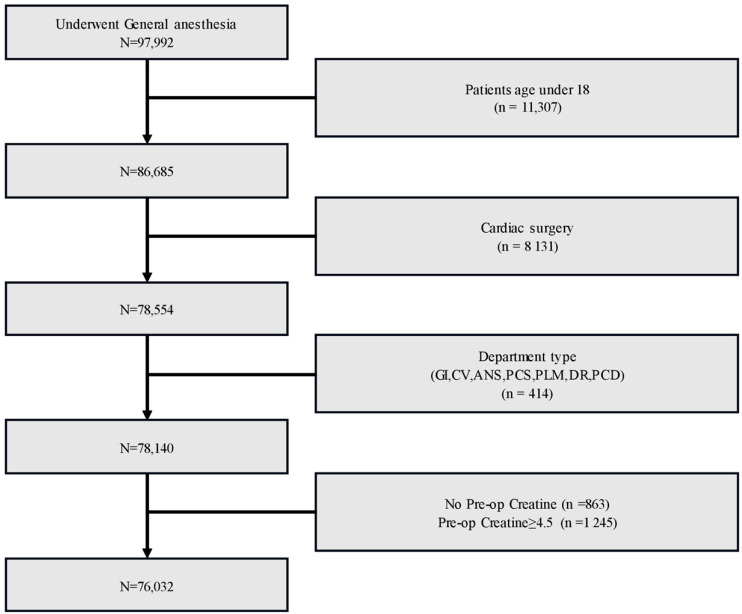
Study flow chart of the internal dataset.

This study utilized the data from 76,032 adult patients aged 18 years and older who underwent non-cardiac surgery at Asan Medical Center (AMC) from March 2019 to February 2021, with the data extracted from the hospital’s Electronic Health Record (EHR) system. In addition, we utilized the open dataset VitalDB for the external validation of the model. Out of the total of 6388 patients in VitalDB, we extracted data for 5512 patients to use as the external validation dataset for our prediction model (Figure S1). The study received approval from the Institutional Review Board (IRB) of Asan Medical Center (IRB No. 2024-0060), and informed consent from participants was waived based on the retrospective collection and secondary utilization of EHR data. We excluded pediatric patients under the age of 18, individuals who underwent cardiac surgery, and those who were diagnosed with end-stage renal disease or had preoperative creatinine levels exceeding 4.5 mg/dL (Figure 1).

### 2.2. Variables for Modeling and Preprocessing Data

The variables employed in the modeling of this study encompassed demographic information, preoperative data, and intraoperative data (Table S1). The demographic data included the patient’s age, gender, body mass index (BMI), and American Society of Anesthesiologists (ASA) class. The preoperative data included preoperative laboratory test results such as the white blood cell, hemoglobin, hematocrit, platelet, sodium, potassium, chloride, total bilirubin, albumin, aspartate aminotransferase (AST), and alanine aminotransferase (ALT) results, along with the estimated glomerular filtration rate (eGFR), glucose level, prothrombin time (PT), activated partial thromboplastin time (aPTT), blood urea nitrogen (BUN) level, creatinine level, and C-reactive protein (CRP) level. The intraoperative data included the arterial blood pressure values (systolic blood pressure (SBP), diastolic blood pressure (DBP), and mean blood pressure (MBP)) measured through arterial catheterization, non-invasive blood pressure values (SBP, DBP, and MBP), estimated blood loss, anesthetic time, and surgery time. In our study, anesthetic time refers to the total duration from the induction of anesthesia to the moment of extubation and the patient’s return to consciousness. Surgery time denotes the period from the initial skin incision by the surgeon to the completion of the surgery. The intraoperative blood pressure data were employed as input variables for the model by computing the maximum, minimum, mean, and standard deviation values of the blood pressure measurements, along with the sum of the changes in the blood pressure differences. Intraoperative monitoring measured blood pressure every 3 to 5 min using non-invasive methods, while arterial catheterization provided continuous real-time blood pressure data. However, the anesthesia records analyzed in our study only documented blood pressure readings at 5 min intervals, so more frequent measurements were not accessible. Intraoperative blood pressure data, which is automatically linked to the electronic medical record, often contains artifacts. We removed these artifacts by using the algorithm presented in our previous study [13]. Blood pressure measurements exceeding the following thresholds were excluded from the analysis: a systolic blood pressure greater than 300 mm Hg or less than 20 mm Hg, diastolic blood pressure greater than 225 mm Hg or less than 5 mm Hg, and systolic blood pressure less than the diastolic blood pressure + 5 mm Hg, in accordance with the criteria proposed in a previous study [13,14]. Since the missing values in the modeling dataset did not exhibit any specific pattern or correlation, we addressed them by inputting the missing values with the mean value of each variable in the dataset (Figures S2 and S3).

### 2.3. Open Dataset for External Validation

In our study, we utilized an open dataset known as VitalDB to externally validate the performance of our prediction model [15]. VitalDB comprises preoperative data from 6388 surgical patients who underwent non-cardiac surgery at Seoul National University Hospital, including intraoperative monitoring parameters. Since AKI, the primary outcome of our study, relies on creatinine blood test results, we chose VitalDB as our external validation dataset because it provides both preoperative and postoperative blood laboratory test data. Furthermore, VitalDB is publicly accessible, allowing researchers to download it from the internet without the need for institutional IRB approval. In this regard, it proves valuable as an external validation dataset for a range of clinical studies.

### 2.4. Primary Outcome

The primary outcome in our study was the occurrence of AKI within 7 days following surgery. AKI is defined as either a 1.5-fold increase in the creatinine level from the preoperative baseline or a rise of 0.3 mg/dL over a 48 h period, as measured within the first 7 days after surgery, in accordance with the definition from the Kidney Disease Improving Global Outcomes (KDIGO) guidelines. If no creatinine level was measured postoperatively, it was assumed that an AKI had not occurred.

### 2.5. Modeling and Model Evaluation

In this study, the dataset variables used for modeling were categorized into demographic, preoperative, and intraoperative datasets. We compared the performances of models based on the individual datasets as well as various combinations of dataset configurations. We divided the datasets for modeling into training, testing, and validation datasets in a ratio of 6:2:2. In order to mitigate any potential overfitting of the model performance stemming from the specific composition of the training dataset, we employed the bootstrapping method for model evaluations by resampling each dataset multiple times. We here present the results of evaluations of the models in the form of the mean values and statistical confidence intervals for the performance outcomes of the trained models on each resampled dataset. In our study, we employed four predictive modeling methods: logistic regression (LR), random forest (RF), gradient boosting machine (GBM), and a simple deep neural network (DNN) with five layers. We configured the hyperparameters for our modeling techniques as follows. For LR, default values were used for the L2 penalty and “lbfgs” solver. For RF and GBM, we used a grid search approach to optimize the hyperparameters. Our DNN model comprised five hidden layers, along with the incorporation of batch normalization and a dropout rate of 0.5. The dense layers utilized rectified linear unit (ReLU) activation functions, and the final output layer employed a sigmoid activation function for binary classification. We applied a learning rate of 0.001, employed binary cross-entropy as the loss function, and utilized the adam optimizer. In our study, we primarily assessed the prediction model’s performance using two key metrics: the area under the receiver operating characteristic (AUROC) curve and area under the precision–recall curve (AUPRC). We employed the DeLong test to establish the statistical significance of the performance differences. Furthermore, we identified and compared the crucial variables influencing the performance of each model using Shapley additive explanation (SHAP) values.

### 2.6. Statistical Analysis and Modeling Tools

To statistically describe the dataset characteristics, the data distributions of continuous variables are depicted using mean and standard deviation values, whereas categorical variables are presented using counts and proportions. To compare continuous variables across datasets, *t*-tests were employed, whereas chi-squared tests were used for categorical variables. The descriptive statistics were obtained using R 3.4.3, whereas ML predictive modeling and model performance evaluations were conducted using Python 3.11.

## 3. Results

### 3.1. Study Data Characteristics

Table 1 presents the results of a comparative analysis of the attributes of research data extracted from two distinct datasets: the AMC data (an internal dataset) and VitalDB data (an external dataset). Initially, there does not seem to be any significant disparities in demographic variables such as age, gender, and BMI between the two datasets (Table 1). Nevertheless, upon closer examination of the surgical department information, it becomes apparent that the external dataset exhibits a higher prevalence of general and thoracic surgeries in contrast to the internal dataset (Table 1). Among the 76,032 adult patients aged 18 years and older who underwent general anesthesia for non-cardiac surgery at AMCs, a total of 2314 (3.1%) patients experienced postoperative AKI. Conversely, for the external validation dataset from VitalDB, which included 5512 patients who did not meet the exclusion criteria, 78 (1.5%) developed postoperative AKI (Table 2). The percentage of patients with a hospital stay of 7 days or more was higher for the external dataset, at 57.2%, in contrast to 38.5% for the internal dataset (Table 2). Mortality within 30 days of surgery did not exhibit a significant difference between the two datasets; however, postoperative in-hospital mortality was higher for the internal dataset, registering at 2.1% compared to 0.9% for the external dataset (Table 2). Additionally, the rate of postoperative intensive care unit (ICU) admissions for the internal dataset was nearly twice as high, standing at 18.3% compared to 9.5% for the external dataset (Table 2). For our internal dataset, we conducted a comparison of clinical outcomes between the AKI and non-AKI groups, and as anticipated, the AKI group exhibited a higher incidence of extended hospital stays, postoperative in-hospital mortality, and postoperative ICU admissions compared to the non-AKI group.

### 3.2. Predictive Performance Results of Internal and External Validations

**Figure 2:**
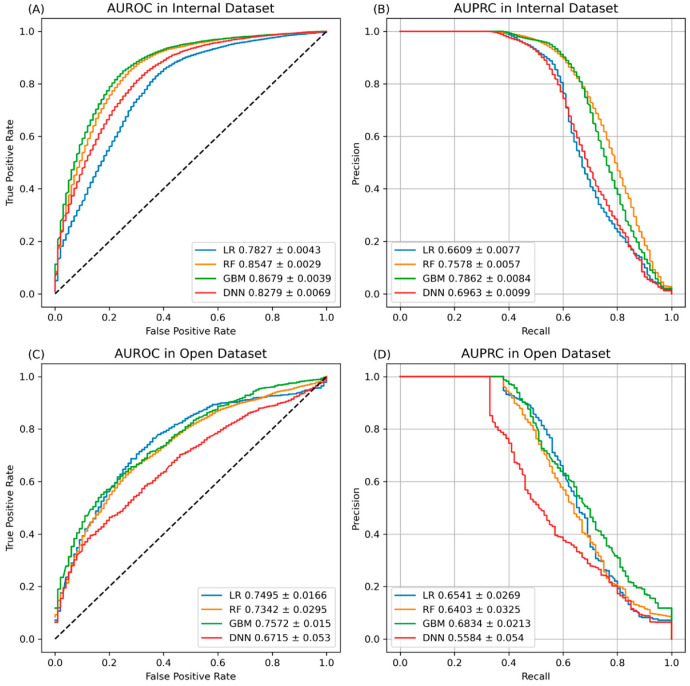
AUROC and AUPRC values of prediction models across various modeling methods using all features in the internal and external datasets. (**A**) AUROC and (**B**) AUPRC values of prediction models across various modeling methods using all features in the internal datasets. (**C**) AUROC and (**D**) AUPRC values of prediction models across various modeling methods using all features in the external datasets. AUROC and AUPRC values are represented as 95% confidence intervals. AUROC, area under receiver operating characteristic; AUPRC, area under precision–recall curve; LR, logistic regression; RF, random forest; GBM, gradient boosting machine; DNN, deep neural network.

The internal validation results for the AKI prediction model showed that the GBM algorithm had the highest prediction performance when using all the datasets, with an AUROC of 0.868, AUPRC of 0.786, and F1-score of 0.723 (Figure 2 and Table 3). The GBM algorithm also had the highest performance when utilizing the intraoperative dataset, with an AUROC of 0.816 and AUPRC of 0.715, as compared to the prediction model performances when using the demographic and preoperative datasets (Table 3). The external validation results for the AKI model on the VitalDB dataset showed the highest prediction performance, with an AUROC of 0.769 and AUPRC of 0.696, was achieved by the GBM algorithm when using the preoperative dataset (Table 4). Furthermore, the LR algorithm exhibited the highest prediction performance when utilizing the entire dataset, with an F1-score of 0.627 (Table 4).

### 3.3. Feature Importance

**Figure 3:**
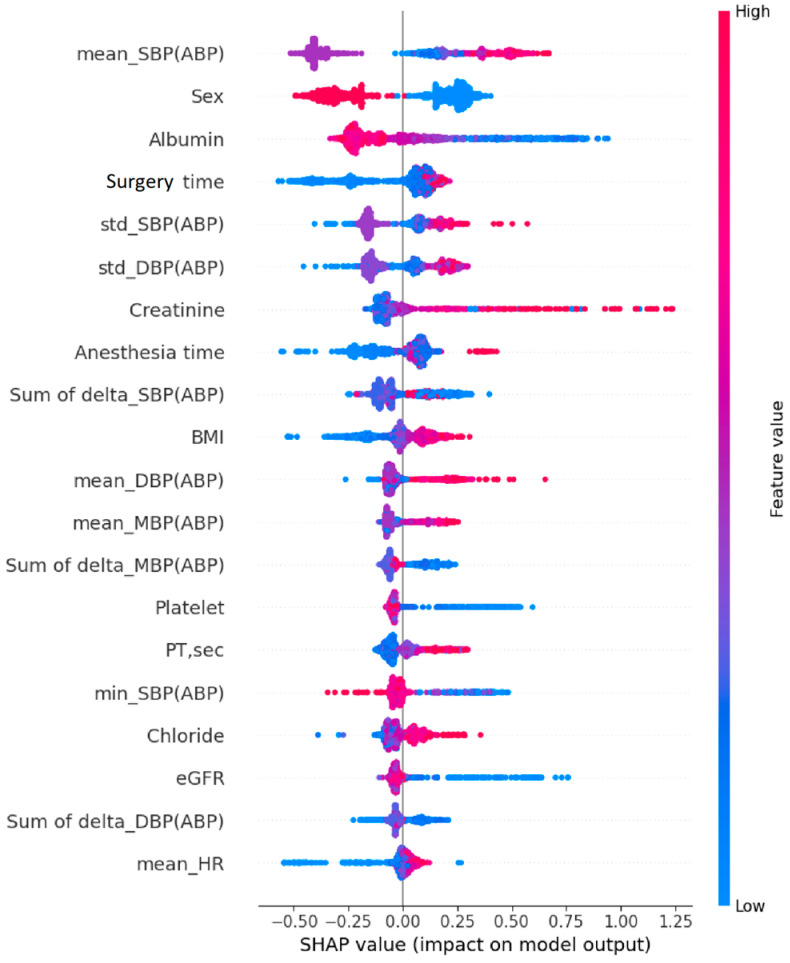
Feature importance of the GBM model for predicting postoperative AKI using SHAP values. GBM, gradient boosting machine; AKI, acute kidney injury; SHAP, Shapley additive explanation; SBP, systolic blood pressure; ABP, arterial blood pressure; DBP, diastolic blood pressure; BMI, body mass index; PT, prothrombin time; std, standard deviation; min, minimum.

An analysis of the feature importance of the AKI prediction model using SHAP values on the internal dataset showed that total surgery time and intraoperative blood pressure data were highly significant, whereas the gender status from the demographic data, and albumin and creatinine levels in the preoperative laboratory test data, played pivotal roles in the prediction performance (Figure 3).

## 4. Discussion

In our study, we developed a model for predicting postoperative AKI by utilizing both preoperative and intraoperative data. We subsequently conducted an external validation of its performance using a publicly available dataset from a distinct institution, thus highlighting the generalizability of our predictive model. Furthermore, by utilizing a substantial EHR dataset, our study demonstrated the potential to predict the occurrence of AKI—a significant postoperative complication. We achieved this using automatically generated EHR data that could be seamlessly integrated with a hospital’s electronic medical record (EMR) system. This integration would serve the dual purposes of alerting medical personnel to the risk in advance, thereby facilitating the provision of intensive care to patients at risk, and assisting in optimizing the allocation of hospital resources by appropriately identifying and reclassifying these at-risk patients.

AKI is a major postoperative complication, contributing significantly to prolonged hospital stays, increased postoperative morbidity, and mortality in patients [1,3,4,16,17,18]. The incidence of postoperative AKI varies widely based on the type of surgery, with surgical factors being some of the most common causes [2]. Whereas AKI is generally less frequent in non-cardiac surgeries as compared to cardiac procedures, its occurrence in the former can present greater prediction challenges, potentially leading to delayed detection and treatment, and consequently worsening the patient’s prognosis. Postoperative AKI is associated with prolonged postoperative mechanical ventilation, extended intensive care hospitalization, and increased treatment requirements, all of which negatively impact the patient’s postoperative outcome [1,3,4,6,7,16,17,18,19,20,21]. Therefore, the ability to predict an AKI before surgery is crucial for efficiently allocating hospital resources to at-risk patients and directing medical resources to high-risk patients, ultimately leading to improved patient outcomes [8,9,22]. Previous studies have explored the use of ML algorithms to predict the occurrence of postoperative AKI [8,9,11,23,24,25,26,27]. Lei et al. aimed to predict the risk of postoperative AKI in non-cardiac surgery by utilizing preoperative and intraoperative blood pressure data [9]. Nevertheless, a limitation of this study was its reliance solely on data from a single institution. This limitation hindered the ability to assess the generalizability of their findings to other healthcare facilities and also made it challenging to evaluate the potential overfitting issue in the prediction model. Overfitting is a common concern in AI research, particularly in studies that solely rely on data from a single institution. Therefore, for the developed ML prediction model to hold significance in terms of reproducibility and generalizability, it needed to undergo validation using data from other institutions to assess its predictive performance in an external context. An external validation of the effectiveness of our ML prediction model was performed by utilizing the VitalDB dataset, which is an open dataset containing information on surgical patients [15].

One noteworthy aspect of our study was our approach to validation. Unlike previous studies that predominantly relied on single-institution data for internal validation, we took a different path by extensively validating our model’s performance against external institutional datasets. Whereas it is recognized that external validation may yield slightly less predictive accuracy compared to internal validation, our research underscored that the predictive performance achieved through external validation remained notably strong, particularly when compared to findings from prior studies. This pivotal finding highlighted our study’s distinctive contribution in addressing a common limitation observed in AI model research, namely the limited generalizability of models trained on data from a single institution, which results in their suboptimal performance on datasets from other institutions.

One of the paramount considerations in AI prediction research involving medical data is to ensure that the developed model avoids overfitting to the training data. This step is crucial for enhancing the model’s applicability in real-world clinical scenarios, making the outcomes of such studies more practically relevant [28,29]. Consequently, we believe that external validation, employing data from external sources, is an essential and vital phase in medical AI prediction research. Nevertheless, acquiring data from external organizations often proves to be a substantial challenge. This difficulty stems from the various data regulations governing these organizations, which impose stringent restrictions on the exchange and sharing of large-scale data across different time periods. As a result, a practical alternative is to evaluate predictive models using single-institution data on publicly available open datasets. This approach offers a valuable means of conducting external validation in AI prediction research within real-world healthcare settings.

Our study had several strengths compared to prior research. First, we considered not only the basic statistical metrics of intraoperative blood pressure, which is a critical parameter, but also changes in blood pressure data. Additionally, we incorporated factors like intraoperative blood loss and surgery time information into our prediction model. Consequently, we enhanced the postoperative AKI prediction performance, achieving an AUC of 0.87, which surpassed the previous study’s AUC of 0.82 [8,9]. The external validation also demonstrated strong predictive performance, with an AUC of 0.769.

Our study had several limitations that warrant consideration in future research. First, we did not use the complete intraoperative blood pressure (BP) dataset for the predictive modeling; instead, we utilized statistical summaries of the BP data. Therefore, the modeling process did use all the BP data but substituted a statistical representation derived from predictive modeling using the complete blood pressure dataset. Future research should emphasize the application of predictive modeling techniques such as transformers and recurrent neural networks (RNN), which are known for their effectiveness in handling time series data like blood pressure readings. The goal should be to develop models capable of capturing all of the blood pressure data. Moreover, it is imperative to validate the applicability of predictive models across diverse real-world organizations using a more comprehensive set of publicly available datasets. Prospective studies are also essential to assess the practical value of these predictive models when deployed in real clinical settings. Ultimately, such prospective investigations can offer valuable insights into the clinical utility of AI predictive models.

## 5. Conclusions

In conclusion, we successfully developed and implemented a prediction model for postoperative AKI in adult patients undergoing non-cardiac surgery by using a dataset that could automatically be extracted from an EHR system. Furthermore, we conducted an external validation of our prediction model using a separate institutional dataset, which was an open dataset, thereby confirming the reproducibility and generalizability of our model. In addition, our study underscored the viability of open datasets as a valuable alternative to third-party datasets for the external validation of prediction models in the field of medical AI research. As additional diverse datasets become available in the future, it is anticipated that more researchers will leverage them for validating a range of AI prediction models.

## Figures and Tables

**Table 1:** Characteristics of the internal and external datasets.

	Internal Dataset			Open Database
	Total (n = 76,032)	AKI (n = 2314)	Non-AKI (n = 73,718)	Total (n = 5512)
Demographic data				
Age, years	55.3 ± 15.2	59.0 ± 13.4	55.2 ± 15.3	58.2 ± 14.0
Sex (female)	34,491 (45.4)	1647 (71.2)	32,844 (44.6)	2766 (50.2)
BMI (kg/m^2^)	24.2 ± 3.7	24.7 ± 3.8	24.2 ± 3.7	23.4 ± 3.6
ASA				
1	7826 (10.3)	149 (6.4)	7677 (10.4)	1612 (29.2)
2	60,450 (79.5)	1439 (62.2)	59,011 (80.1)	3337 (60.5)
3	7092 (9.3)	595 (25.7)	6497 (8.8)	516 (9.4)
≥4	661 (0.8)	130 (5.6)	531 (0.7)	47 (8.5)
Surgery department				
GS	32,092 (42.2)	1347 (58.2)	30,745 (41.7)	4272 (77.5)
URO	7828 (10.3)	677 (29.3)	7151 (9.7)	116 (2.1)
OBY	7785 (10.2)	46 (1.9)	7739 (10.5)	197 (3.6)
OS	7237 (9.5)	78 (3.4)	7159 (9.7)	-
ENT	5699 (7.5)	32 (1.4)	5667 (7.7)	-
NS	5149 (6.8)	20 (0.9)	5129 (7.0)	-
CS	4409 (5.8)	93 (4.0)	4316 (5.9)	927 (16.8)
PS	2691 (3.5)	15 (0.6)	2676 (3.6)	-
OPH	2254 (3.0)	1 (0.0)	2253 (3.1)	-
DNT	878 (1.2)	5 (0.2)	873 (1.2)	-
DER	10 (0.0)	0 (0.0)	10 (0.0)	-
Preoperative laboratory results				
White blood cell, 10^3^/μL	6.5 ± 2.6	6.6 ± 3.3	6.5 ± 2.5	6.2 ± 2.4
Hemoglobin, g/dL	12.9 ± 1.8	12.3 ± 2.3	12.9 ± 1.8	12.9 ± 1.9
Sodium, mmol/L	140.2 ± 2.6	139.8 ± 3.5	140.3 ± 2.5	140.1 ± 2.8
Platelet, 10^3^/μL	241.8 ± 75.9	208.0 ± 99.6	242.9 ± 74.8	242.87 ± 83.4
Potassium, mmol/L	4.2 ± 0.4	4.2 ± 0.5	4.3 ± 0.4	4.2 ± 0.4
Chloride, mmol/L	103.7 ± 2.9	103.5 ± 3.9	103.7 ± 2.9	103.3 ± 4.0
Total bilirubin, mg/dL	0.6 ± 1.6	1.4 ± 3.9	0.6 ± 1.4	0.7 ± 1.7
BUN, mg/dL	15.3 ± 6.5	17.7 ± 10.1	15.2 ± 6.4	14.8 ± 6.9
Creatinine, mg/dL	0.8 ± 0.3	1.0 ± 0.6	0.8 ± 0.3	0.8 ± 0.3
Albumin, g/dL	3.8 ± 0.5	3.5 ± 0.6	3.8 ± 0.5	4.1 ± 0.5
AST, IU/L	24.8 ± 49.2	40.0 ± 186.1	24.3 ± 37.6	31.1 ± 140.0
ALT, IU/L	22.4 ± 40.8	32.3 ± 148.2	22.1 ± 32.1	29.1 ± 95.2
Hematocrit, %	38.9 ± 5.0	36.8 ± 6.6	38.9 ± 4.9	37.4 ± 6.1
eGFR, mL/min/1.73 m^2^	92.5 ± 19.9	85.0 ± 26.6	92.7 ± 19.5	86.7 ± 26.4
Glucose, mg/dL	115.2 ± 40.4	122.9 ± 49.0	114.9 ± 40.1	115.8 ± 41.7
PT, INR	1.0 ± 0.2	1.1 ± 0.4	1.0 ± 0.2	1.0 ± 0.2
aPTT, s	27.6 ± 4.4	29.5 ± 8.5	27.6 ± 4.2	32.9 ± 8.6
CRP, mg/dL	1.2 ± 3.4	1.9 ± 4.5	1.1 ± 3.3	1.2 ± 3.6
Intraoperative data				
EBL, mL	112.9 ± 232.4	145.0 ± 649.4	23.3 ± 1360.1	365.86 ± 1176.3
Anesthesia time, min	171.5 ± 128.5	301.9 ± 233.4	167.4 ± 121.6	203.9 ± 114.0
Surgery time, min	126.0 ± 114.3	241.8 ± 210.7	122.4 ± 108.0	140.10 ± 100.3

Data represent mean ± standard deviation, median (interquartile range), or number (percentage). AKI, acute kidney injury; BMI, body mass index; ASA, American Society of Anesthesiologists classification; GS, general surgery; URO, urology; OBY, obstetrics and gynecology; OS, orthopedic surgery; ENT, ear, nose, and throat surgery; NS, neurosurgery; CS, cardiothoracic surgery; PS, plastic surgery; OPH, ophthalmology; DNT, dental surgery; DER, dermatology; BUN, blood urea nitrogen; AST, aspartate aminotransferase; ALT, alanine aminotransferase; PT, prothrombin time; aPTT, activated partial thromboplastin time; EBL, estimated blood loss.

**Table 2:** Clinical outcomes in the internal and external datasets.

Clinical Outcome	Internal Dataset			Open Database
	Total (n = 76,032)	AKI (n = 2314)	Non-AKI (n = 73,718)	Total (n = 5512)
Acute kidney injury, n (%)	2314 (3.1)	-	-	78 (1.4)
Length of hospital stay (days)	9.0 ± 38.3	21.9 ± 34.5	8.6 ± 38.3	10.3 ± 13.7
Length of hospital stay ≥ 7 days, n (%)	29,280 (38.5)	1651 (71.3)	27,629 (37.5)	3153 (57.2)
In-hospital death, n (%)	1595 (2.1)	144 (6.2)	1201 (1.6)	47 (0.9)
30-day mortality, n (%)	270 (0.4)	54 (2.3)	216 (0.3)	28 (0.5)
Postoperative ICU care, n (%)	7217 (9.5)	704 (30.4)	6513 (9.8)	1008 (18.3)
Length of ICU stay (days)	0.7 ± 6.7	3.8 ± 13.9	0.6 ± 6.4	0.49 ± 3.3

Data represent mean ± standard deviation, median (interquartile range), or number (percentage). AKI, acute kidney injury; ICU, intensive care unit.

**Table 3:** Predictive performances of machine learning techniques for predicting postoperative AKI in the internal dataset using combinations of various features.

Features	Model	AUROC	AUPRC	F1-Score
Demographic data	LR	0.6942 ± 0.0038	0.5714 ± 0.0070	0.3981 ± 0.0048
	RF	0.6549 ± 0.0048	0.5165 ± 0.0057	0.4931 ± 0.0053
	GBM	0.7137 ± 0.0038	0.5906 ± 0.0077	0.4573 ± 0.0074
	DNN	0.6794 ± 0.0092	0.5060 ± 0.0140	0.2919 ± 0.042
Preoperative data	LR	0.6795 ± 0.0043	0.5942 ± 0.0080	0.4184 ± 0.0059
	RF	0.7224 ± 0.0043	0.6375 ± 0.0061	0.5023 ± 0.0052
	GBM	0.7280 ± 0.0040	0.6468 ± 0.0066	0.5072 ± 0.0047
	DNN	0.6281 ± 0.0161	0.5218 ± 0.0145	0.3707 ± 0.0290
Intraoperative data	LR	0.7449 ± 0.0039	0.6267 ± 0.0066	0.4963 ± 0.0042
	RF	0.7221 ± 0.0034	0.5983 ± 0.0067	0.4595 ± 0.0051
	GBM	0.8161 ± 0.0037	0.7146 ± 0.0082	0.6562 ± 0.0069
	DNN	0.8105 ± 0.0070	0.6749 ± 0.0102	0.6539 ± 0.0109
All features	LR	0.7827 ± 0.0043	0.6609 ± 0.0077	0.5449 ± 0.0056
	RF	0.8547 ± 0.0029	0.7578 ± 0.0057	0.7101 ± 0.0058
	GBM	0.8679 ± 0.0039	0.7862 ± 0.0084	0.7226 ± 0.0062
	DNN	0.8279 ± 0.0069	0.6963 ± 0.00099	0.6355 ± 0.0277

Data are presented as means (95% confidence intervals). AKI, acute kidney injury; AUROC, area under receiver operating characteristic; AUPRC, area under precision–recall curve; LR, logistic regression; RF, random forest; GBM, gradient boosting machine; DNN, deep neural network.

**Table 4:** Predictive performance of machine learning techniques for predicting postoperative AKI in the external dataset using combinations of various features.

Features	Model	AUROC	AUPRC	F1-Score
Demographic data	LR	0.6815 ± 0.0123	0.5491 ± 0.0214	0.4028 ± 0.0063
	RF	0.6031 ± 0.0122	0.4442 ± 0.0161	0.4176 ± 0.0179
	GBM	0.6995 ± 0.0102	0.5900 ± 0.0177	0.5042 ± 0.0153
	DNN	0.6600 ± 0.0108	0.5079 ± 0.0172	0.3171 ± 0.0360
Preoperative data	LR	0.6808 ± 0.0114	0.6443 ± 0.0125	0.4211 ± 0.0153
	RF	0.6031 ± 0.0122	0.4442 ± 0.0161	0.4176 ± 0.0179
	GBM	0.7693 ± 0.0128	0.6958 ± 0.0.185	0.5118 ± 0.0139
	DNN	0.6839 ± 0.00223	0.5978 ± 0.0239	0.4641 ± 0.0386
Intraoperative data	LR	0.7674 ± 0.0101	0.4027 ± 0.0212	0.0292 ± 0.0009
	RF	0.6499 ± 0.0295	0.4748 ± 0.0262	0.4877 ± 0.0273
	GBM	0.7054 ± 0.0168	0.5836 ± 0.0266	0.5985 ± 0.0116
	DNN	0.5770 ± 0.3780	0.489 ± 0.0420	0.4804 ± 0.0196
All features	LR	0.7495 ± 0.0166	0.6541 ± 0.0269	0.6268 ± 0.0189
	RF	0.7342 ± 0.0295	0.6403 ± 0.0325	0.6098 ± 0.0228
	GBM	0.7572 ± 0.0150	0.6834 ± 0.0213	0.6129 ± 0.0205
	DNN	0.6715 ± 0.0530	0.5584 ± 0.0540	0.5292 ± 0.0303

Data are presented as means (95% confidence intervals). AKI, acute kidney injury; AUROC, area under receiver operating characteristic; AUPRC, area under precision–recall curve; LR, logistic regression; RF, random forest; GBM, gradient boosting machine; DNN, deep neural network.

## Data Availability

The data presented in this study are available on reasonable request from the corresponding author.

## Supplementary Materials

The following supporting information can be downloaded at: https://www.mdpi.com/article/10.3390/jpm14060587/s1. Table S1. Variables for modeling; Figure S1. Study flow chart of open dataset; Figure S2. Distribution plots of missing values (A) Distribution plot of missing values in internal dataset. (B) Distribution plot of missing values in external dataset; Figure S3. Nullity correlation heatmaps (A) Nullity correlation heatmap for internal dataset. (B) Nullity correlation heatmap for external dataset; Figure S4. Distribution plots of various blood pressure variables in internal and external datasets; Figure S5. AUROC and AUPRC values of prediction models across different modeling methods according to the various feature selections (A) Demographic dataset. (B) Preoperative dataset. (C) Intraoperative dataset; Figure S6. SHAP value summary plots generated from the GBM models according to the various feature selections (A) Demographic dataset. (B) Preoperative dataset. (C) Intraoperative dataset.
