# Supplementary material for: Internal and External Validation of Machine Learning Models for Predicting Acute Kidney Injury Following Non-Cardiac Surgery Using Open Datasets
Source: J Pers Med. 2024 May 30;14(6):587. doi: 10.3390/jpm14060587 (PMC11204685; doi:10.3390/jpm14060587)

Supplementary Materials

Internal and External Validation of Machine Learning Models for Predicting Acute Kidney Injury Following Non-cardiac Surgery using Open Datasets

Sang-Wook Lee, M.D. Ph.D.[a,†]; Jaewon Jang, M.S.[b,†]; Woo-Young Seo, Ph.D.[b]; Donghee Lee, M.D.[a]; Sung-Hoon Kim, M.D. Ph.D.[a,*]

Contents

Table S1 Variables for modeling.

Figure S1 Study flow chart of open dataset.

Figure S2 Distribution plots of missing values.

(A) Distribution plot of missing values in internal dataset.

(B) Distribution plot of missing values in external dataset.

Figure S3 Nullity correlation heatmaps.

(A) Nullity correlation heatmap for internal dataset.

(B) Nullity correlation heatmap for external dataset.

Figure S4 Distribution plots of various blood pressure variables in internal and external datasets.

Figure S5 AUROC and AUPRC values of prediction models across different modeling methods according to the various feature selections.

(A) Demographic dataset.

(B) Preoperative dataset.

(C) Intraoperative dataset.

Figure S6 SHAP value summary plots generated from the GBM models according to the various feature selections.

(A) Demographic dataset.

(B) Preoperative dataset.

(C) Intraoperative dataset.

Table S1. Variables for modeling.

Dataset	Categories	Variables
Demographic Dataset	Demographic data	Age, years
		Sex
		BMI (kg/m^2)
		ASA physical status
Preoperative Dataset	Laboratory test results	White blood cell, 10^3/μL
		Hemoglobin, g/dL
		Sodium, mmol/L
		Platelet, 10^3/μL
		Potassium, mmol/L
		Chloride, mmol/L
		Total Bilirubin, mg/dL
		Albumin, g/dL
		AST, IU/L
		ALT, IU/L
		Hematocrit, %
		eGFR, ml/min/1.73 m^2
		Glucose, mg/dL
		PT, INR
		aPTT, sec
		BUN, mg/dL
		Creatinine, mg/dL
		CRP, mg/dL
Intraoperative Dataset	Arterial blood pressure	Maximum, SBP
		Minimum, SBP
		Mean, SBP
		Standard deviation, SBP
		Sum of delta blood pressure, SBP
		Maximum, DBP
		Minimum, DBP
		Mean, DBP
		Standard deviation, DBP
		Sum of delta blood pressure, DBP
		Maximum, MBP
		Minimum, MBP
		Mean, MBP
		Standard deviation, MBP
		Sum of delta blood pressure, MBP
	Noninvasive blood pressure	Maximum, SBP
		Minimum, SBP
		Mean, SBP
		Standard deviation, SBP
		Sum of delta blood pressure, SBP
		Maximum, DBP
		Minimum, DBP
		Mean, DBP
		Standard deviation, DBP
		Sum of delta blood pressure, DBP
		Maximum, MBP
		Minimum, MBP
		Mean, MBP
		Standard deviation, MBP
		Sum of delta blood pressure, MBP
	Surgical information	Estimated blood loss

		Surgery time
		Anesthetic time

BMI, body-mass index; ASA, American society of anesthesiologists classification; AST, aspartate aminotransferase; ALT, alanine aminotransferase; eGFR, estimated glomerular filtration rate; PT, prothrombin time; aPTT, activated partial thromboplastin time; BUN, blood urea nitrogen; CRP, c-reactive protein; SBP, systolic blood pressure; DBP, diastolic blood pressure; MBP, mean blood pressure.

Figure S1. Study flow chart of open dataset.

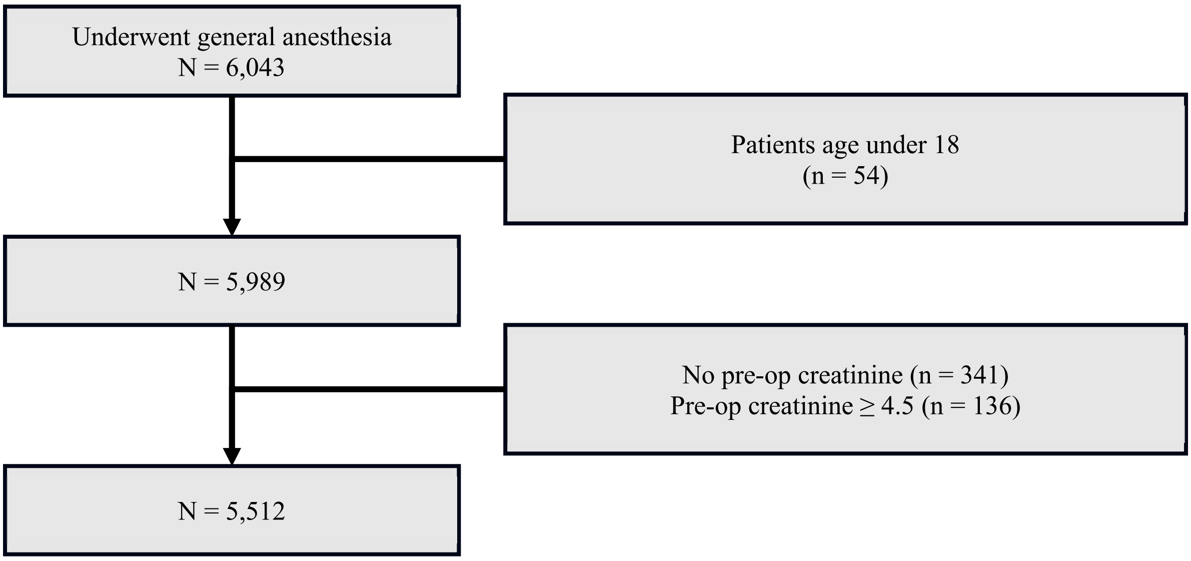

Figure S2. Distribution plots of missing values.

(A) Distribution plot of missing values in internal dataset.

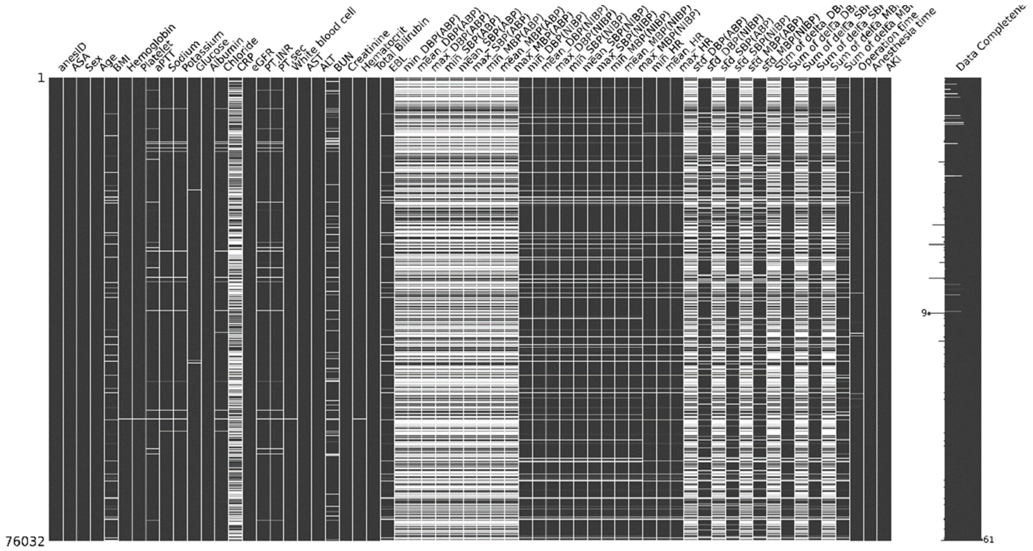

(B) Distribution plot of missing values in open dataset.

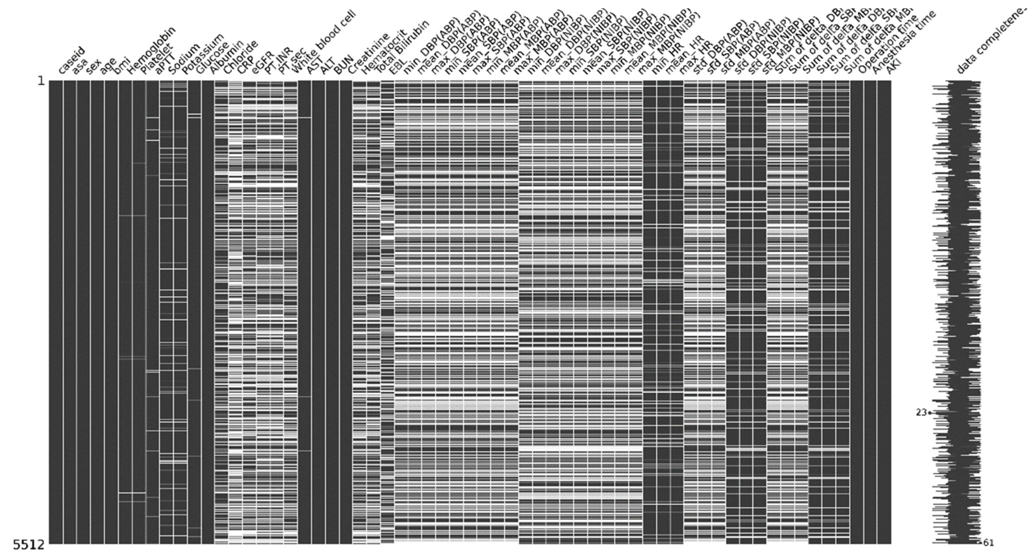

Figure S3. Nullity correlation heatmaps.

(A) Nullity correlation heatmap for internal dataset.

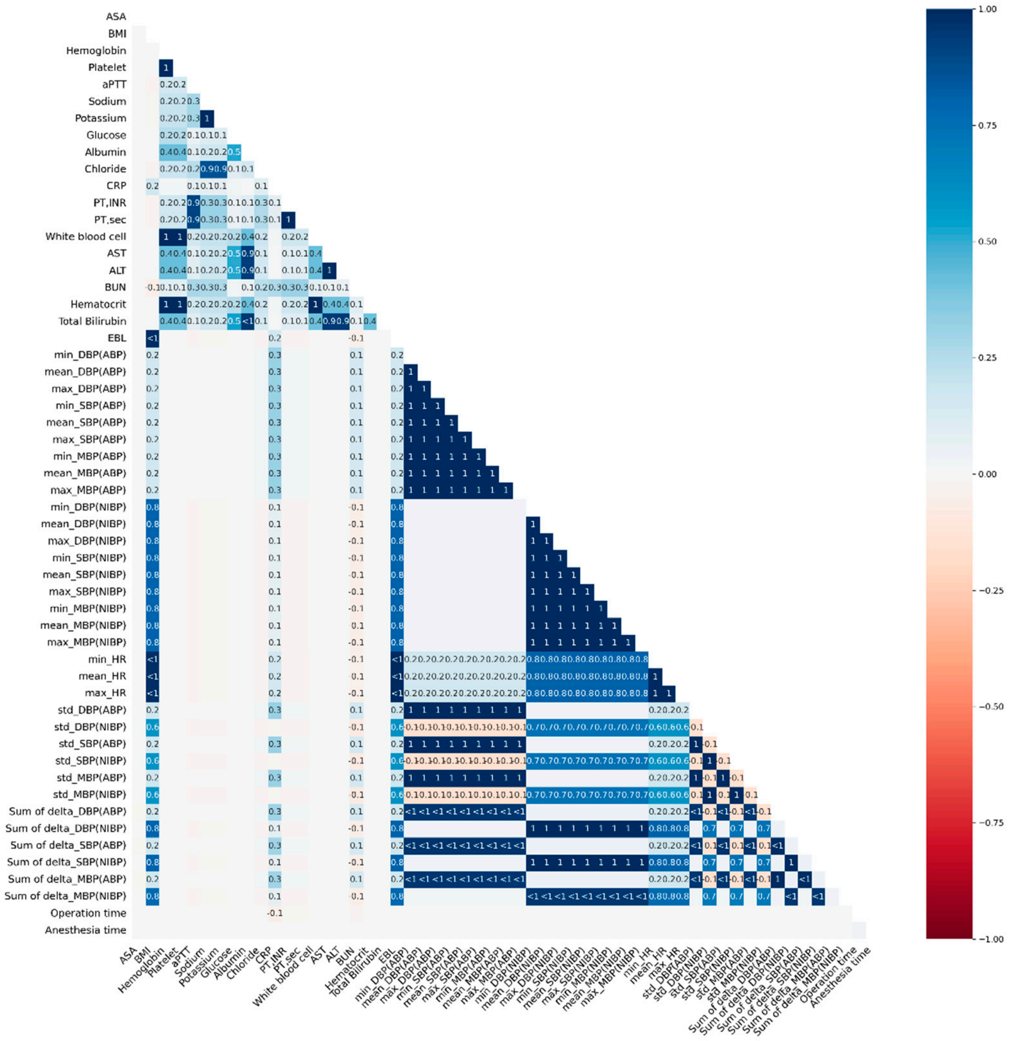

(B) Nullity correlation heatmap for open dataset.

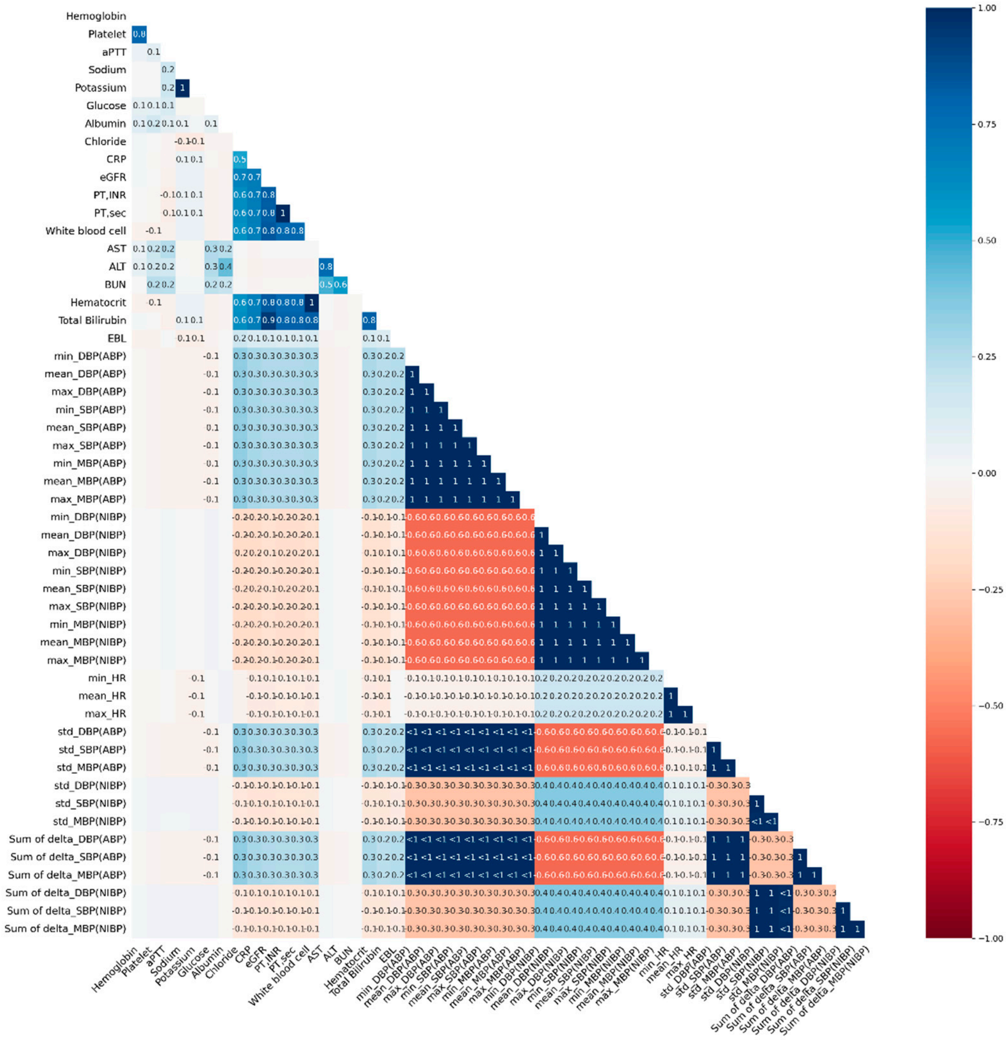

Figure S4. Distribution plots of various blood pressure variables in internal and external dataset.

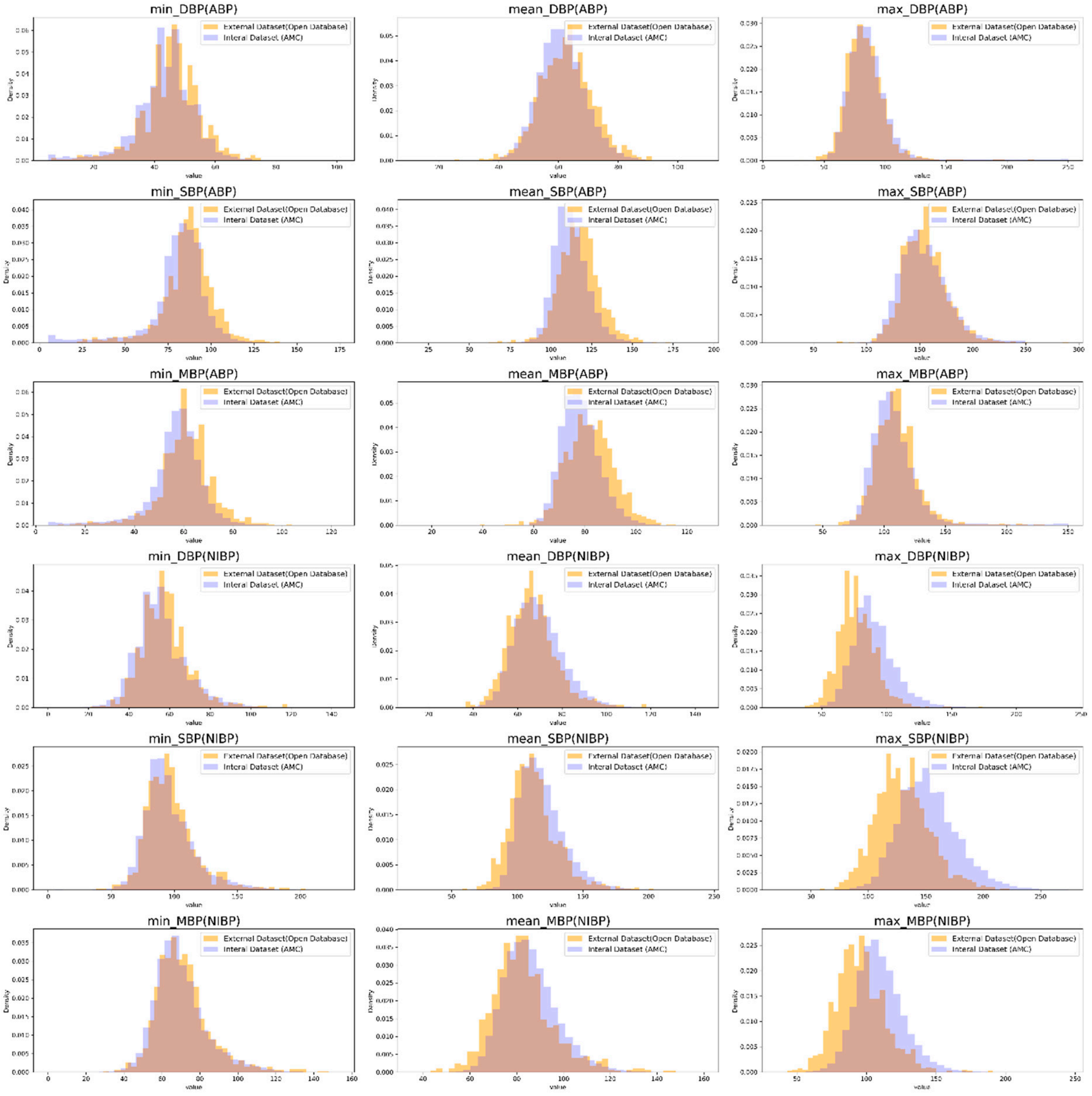

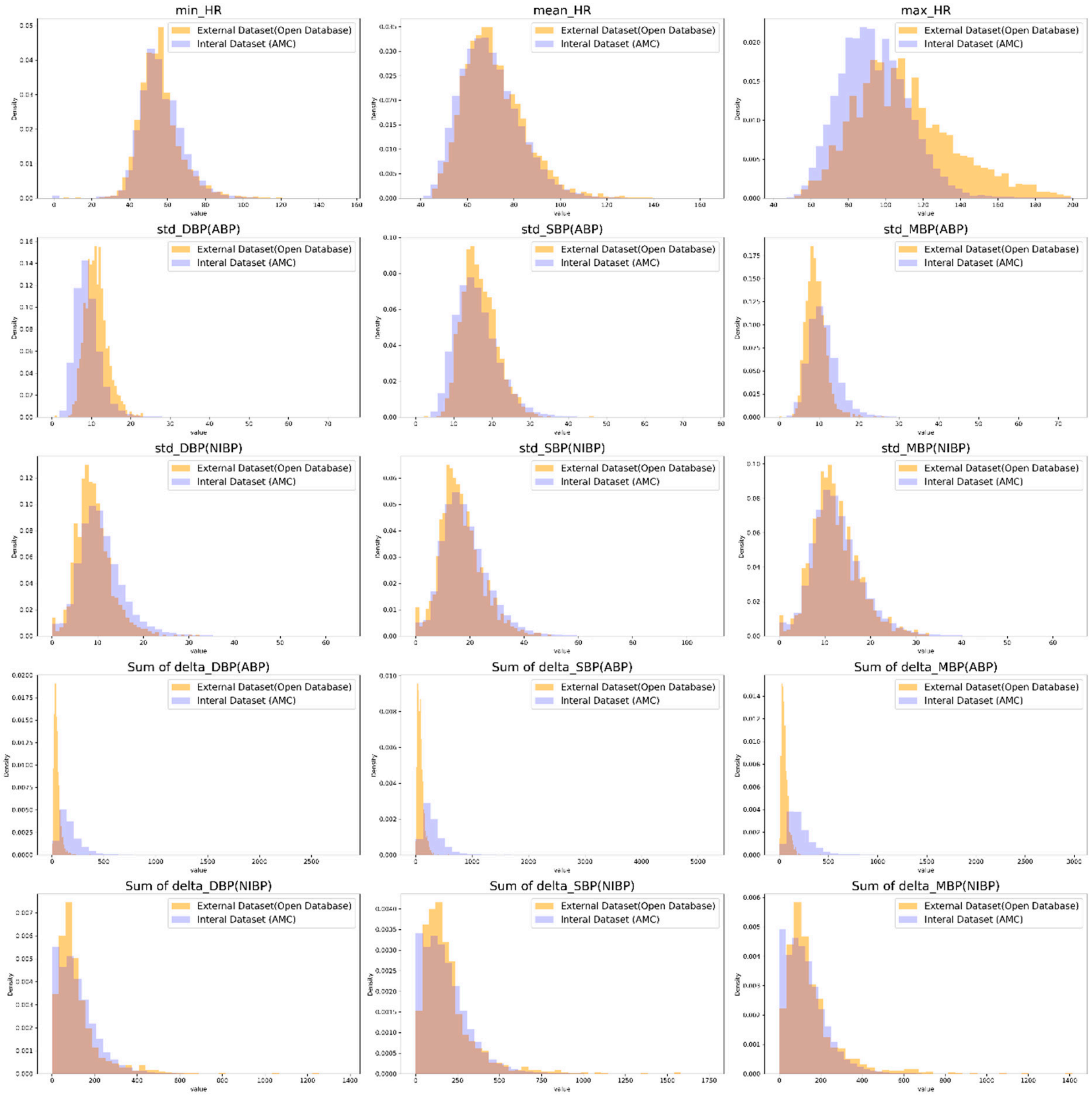
min_HR
mean_HR
max_HR
std_DBP(ABP)
std_SBP(ABP)
std_MBP(ABP)
std_DBP(NIBP)
std_SBP(NIBP)
std_MBP(NIBP)
Sum of delta_DBP(ABP)
Sum of delta_SBP(ABP)
Sum of delta_MBP(ABP)
Sum of delta_DBP(NIBP)
Sum of delta_SBP(NIBP)
Sum of delta_MBP(NIBP)
External Dataset(Open Database)
Interal Dataset (AMC)
Density
value

Figure S5. AUROC and AUPRC values of prediction models across different modeling methods according to the various feature selections.

(A) Demographic dataset.

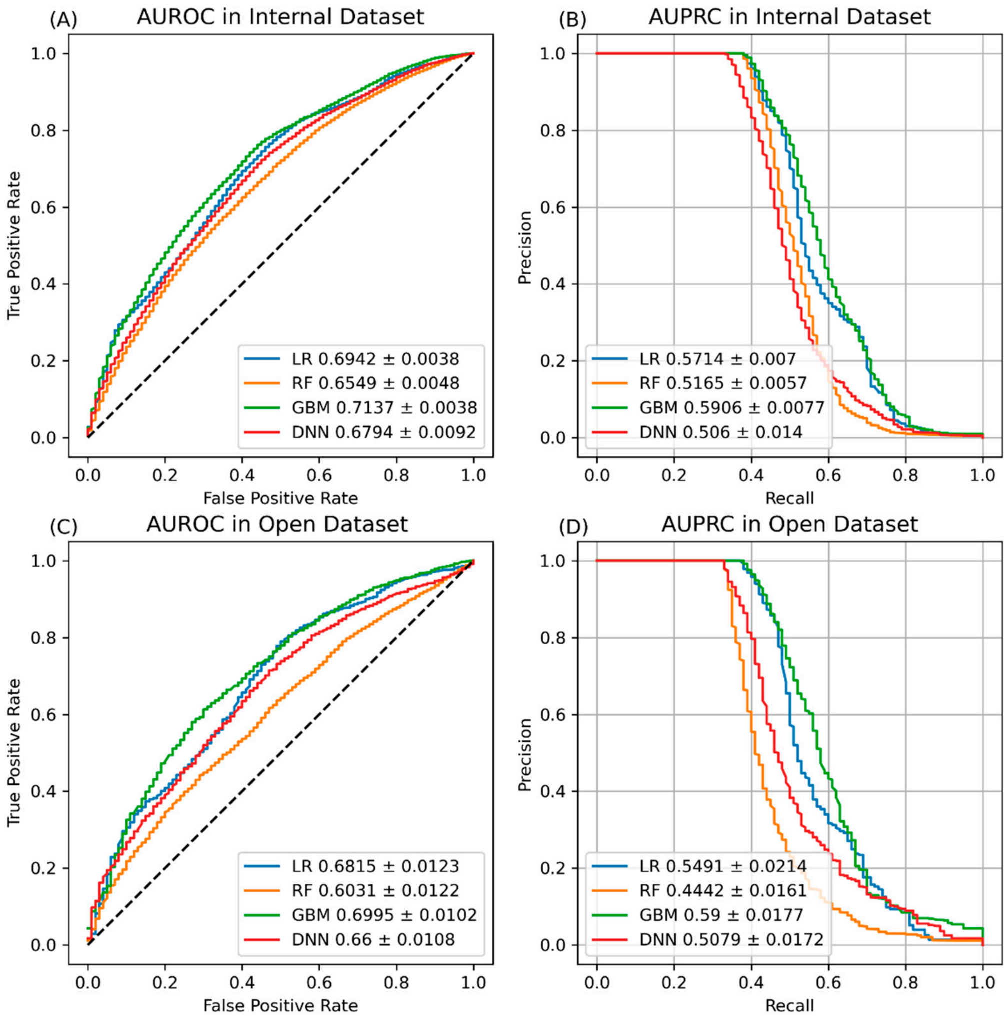

AUROC, area under receiver operating characteristic; AUPRC, area under precision-recall curve.

(B) Preoperative dataset.

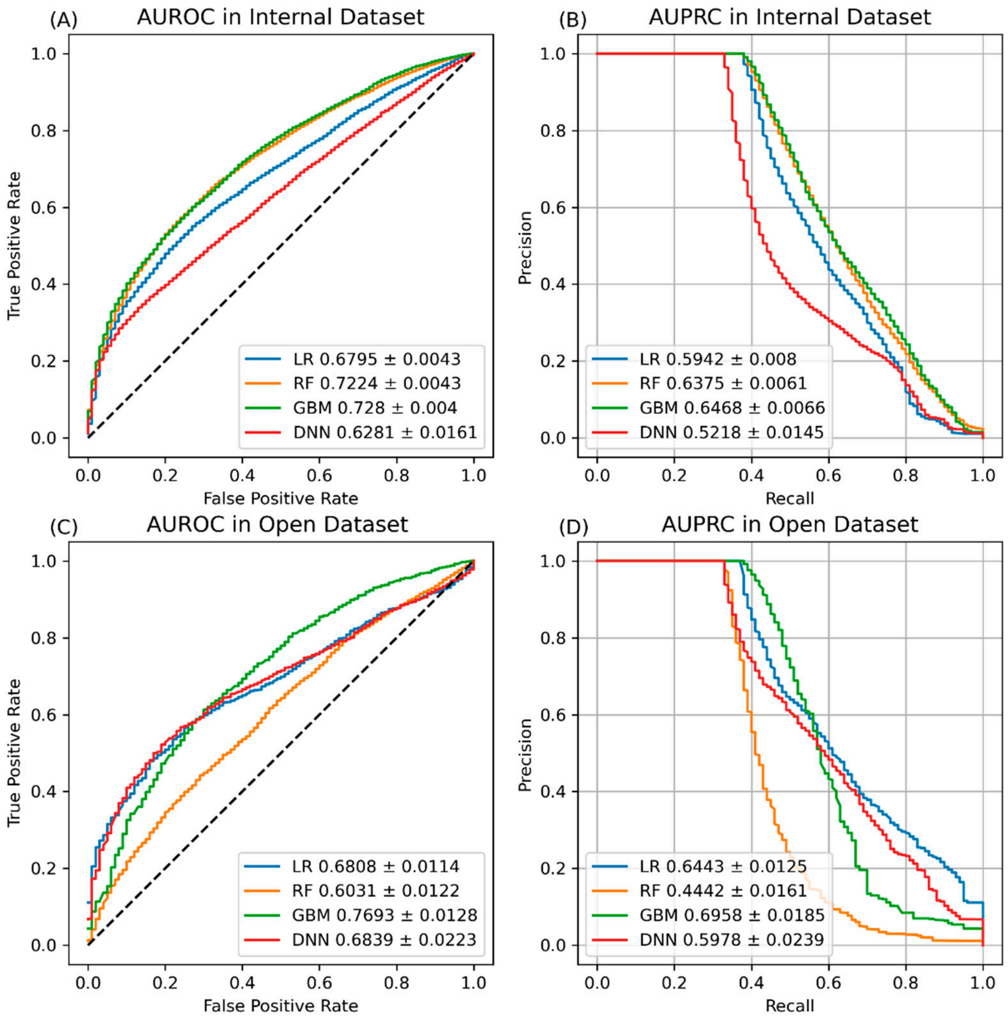

AUROC, area under receiver operating characteristic; AUPRC, area under precision-recall curve

(C) Intraoperative dataset.

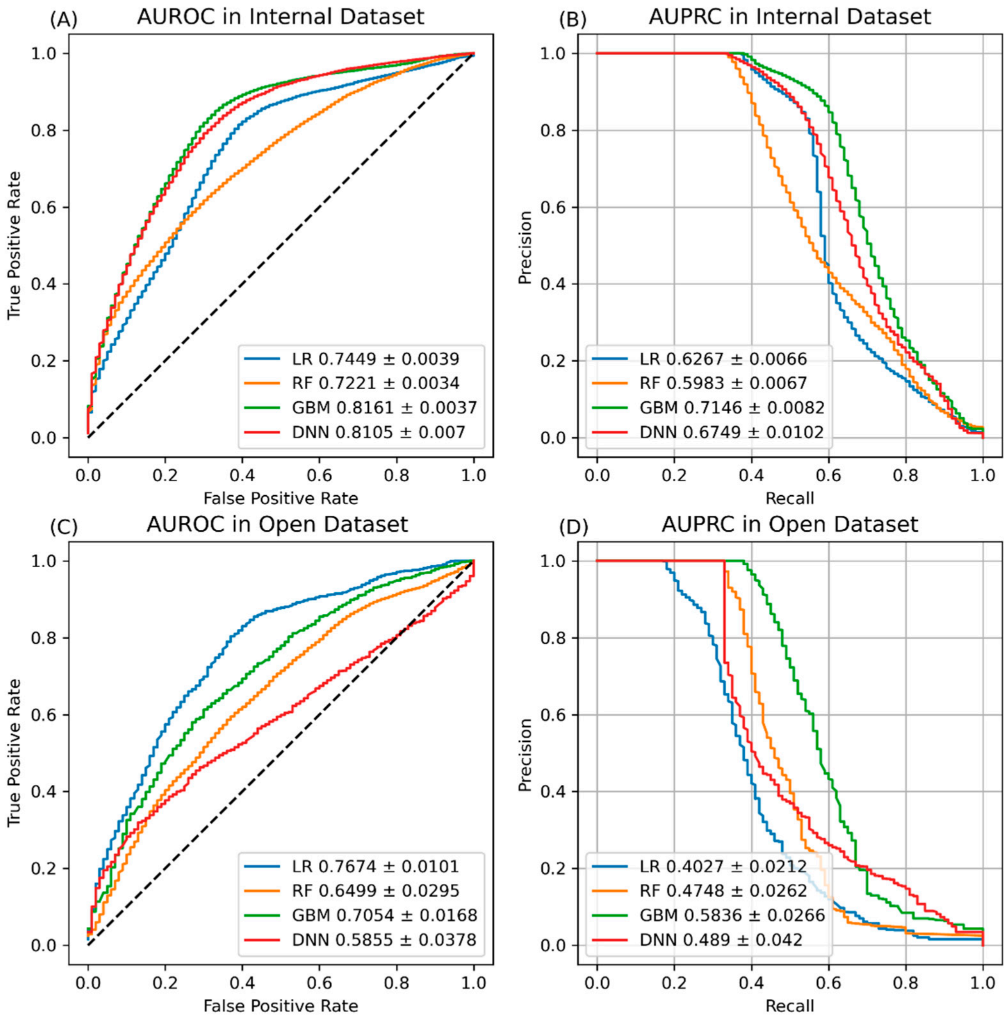

AUROC, area under receiver operating characteristic; AUPRC, area under precision-recall curve

Figure S6. SHAP value summary plots generated from the GBM models according to the various feature selections.

(A) Demographic dataset.

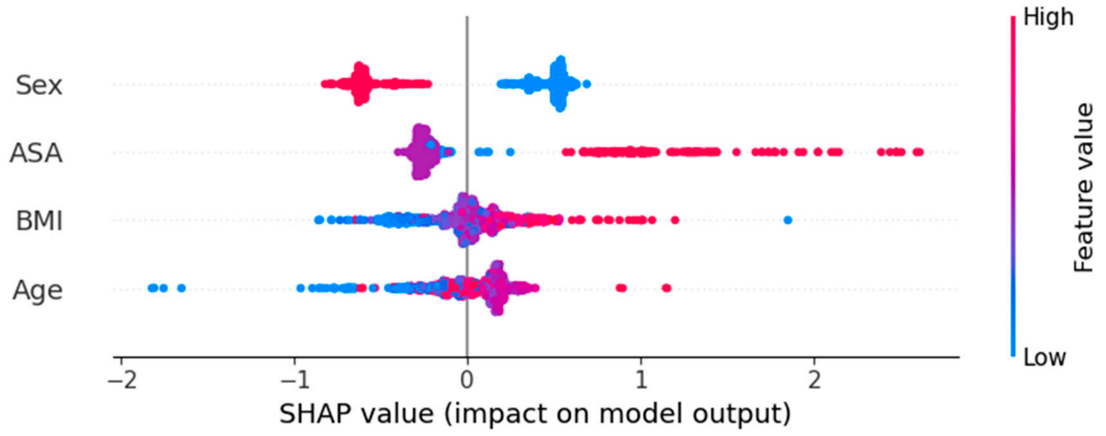

SHAP, shapley additive explanation; GBM, gradient boosting machine.

(B) Preoperative dataset.

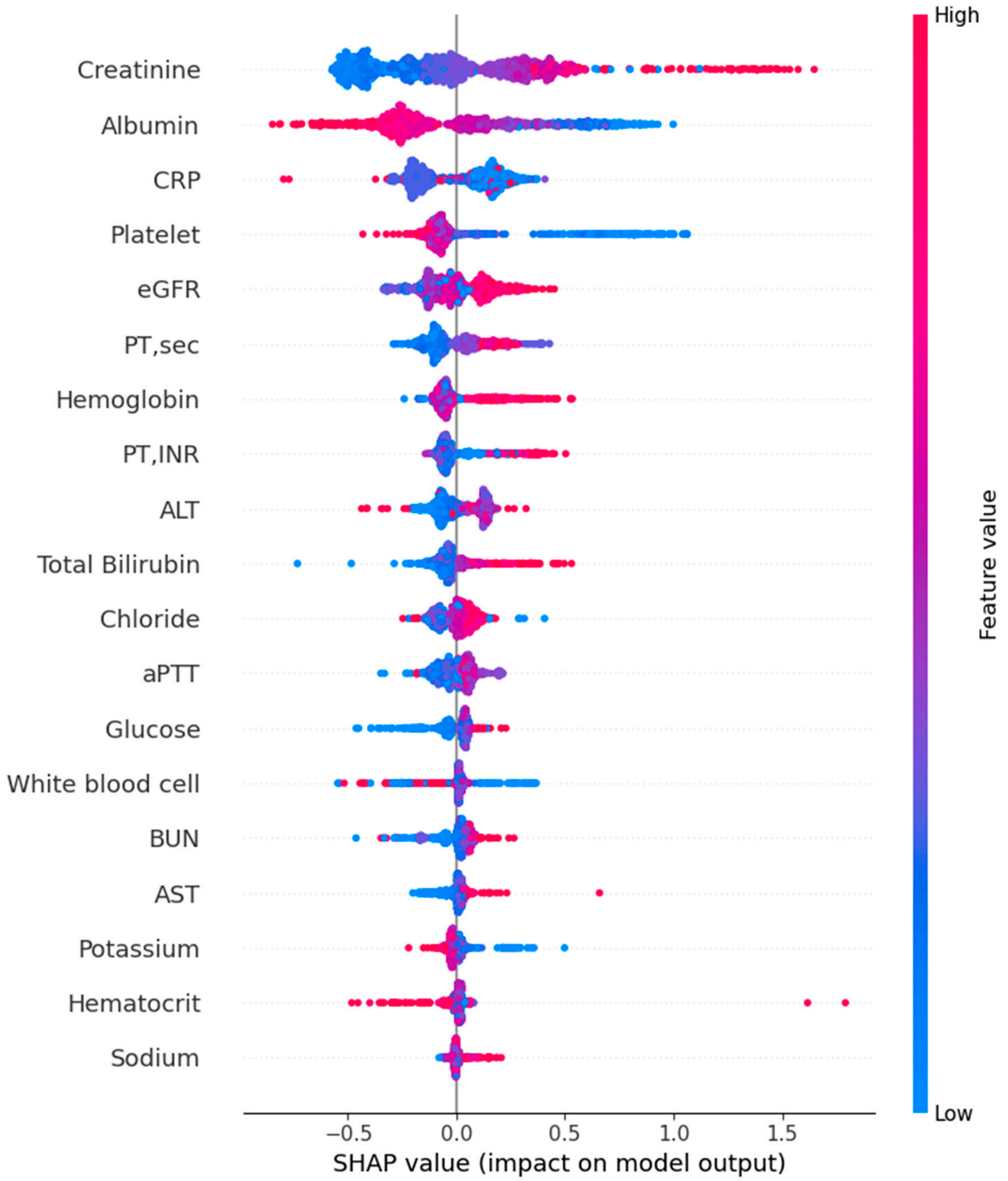

(C) Intraoperative dataset.

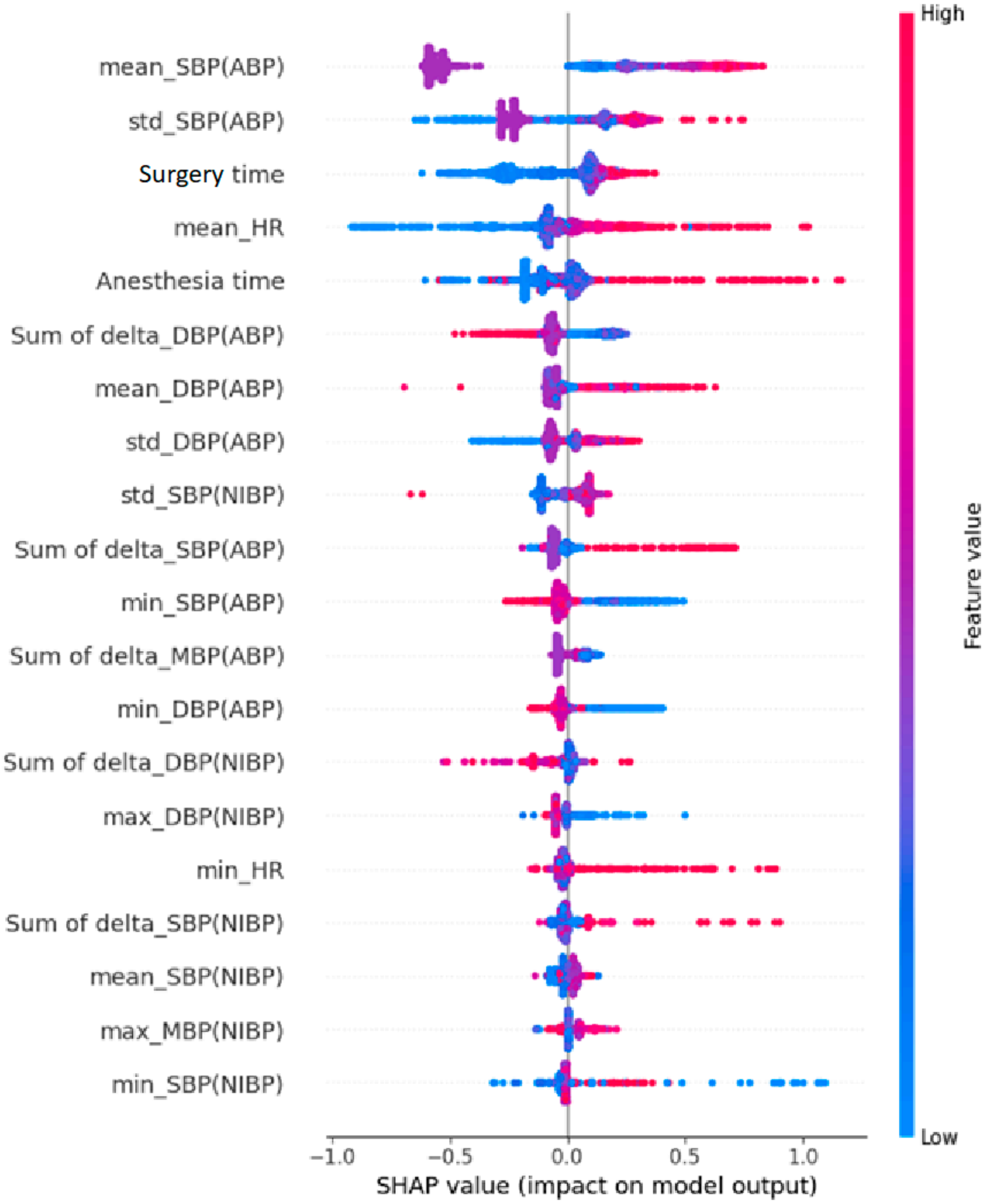

Supplement: Supplementary file 1 [file jpm-14-00587-s001.zip › jpm-3023191-supplementary.pdf]
